# Multi-talented myeloid cells

**DOI:** 10.1093/discim/kyaf015

**Published:** 2025-10-28

**Authors:** Sam Bankole, Lai Guan Ng, Florent Ginhoux

**Affiliations:** University of Edinburgh, The Institute of Regeneration and Repair, Edinburgh, Scotland, UK; Singapore Immunology Network, Agency for Science, Technology and Research, Singapore, Singapore; Paris-Saclay University, Gustave Roussy, INSERM U1015, Villejuif, France

Myeloid cells, including monocytes, macrophages, dendritic cells (DCs), and granulocytes (basophils, eosinophils, mast cells, and neutrophils), are our evolutionarily ancient protectors. With the exception of DCs, these innate immune cells are found across all vertebrates even those without adaptive immunity. They form the first line of defence against pathogens through mechanisms including phagocytosis and production of toxic molecules such as reactive oxygen species. Granulocytes such as basophils, eosinophils, and mast cells have further strategies to combat parasitic infection, a challenge now far less common in industrialized societies.

Through the twentieth century, infectious and parasitic diseases declined with the advent of sterilization techniques, vaccines, and antimicrobials, while immune-mediated disorders such as autoimmunity and allergy became increasingly prevalent [1–3]. In parallel, landmark discoveries in the 1950s—including autoantibodies and the recognition of autoimmunity—drew attention towards the adaptive immune system, with innate cells often regarded as comparatively rudimentary [4]. At the same time, dramatic rises in obesity and its complications reflected the transition to sedentary, hypercaloric lifestyles. In this context, interest in myeloid biology waned—partly due to limited tools for studying these cells compared with approaches available for T and B lymphocytes—and was further overshadowed by an intense focus on adaptive responses.

Over the past couple of decades this perspective has shifted. A renaissance in myeloid cell research underscores the extraordinary versatility and immunological relevance of these cells [4]. Beyond their classical functions in pathogen elimination, inflammation, and antigen presentation, myeloid cells are now recognized as critical regulators of homeostasis, tissue repair, and metabolism, playing important roles in physiology [5]. When dysregulated, they fuel immune-mediated, fibrotic, and degenerative disease. Heterogeneity and plasticity are now recognized as defining features of myeloid cells, reflecting their functional capacity and context-specific roles. This special issue highlights some of the unconventional aspects of myeloid cell biology, showing how these ancient cells remain at the heart of health and disease in modern society.

Arguably, obesity is one of the most important healthcare challenges in high income settings due to its link with increased chronic disease, including diabetes, cardiovascular disease and cancer. Obesity is also associated with poor infection and vaccine responses, yet the underlying mechanisms remain unclear. In this issue, the article by Andrea Woodcock and colleagues shed light on this question by examining how obesity alters DC biology. Using GM-CSF-dependent DCs, they show that antiviral cytokine responses depend on glycolysis to sustain protein translation [6]. When generated from mice fed on a high-fat diet, DCs displayed reduced protein synthesis, impaired interferon and IL-6 production, and diminished antiviral gene expression despite intact NF-κB activation. These findings suggest obesity impairs DC function by constraining metabolic capacity with detrimental consequences for antiviral immunity, and underscores the need to examine how obesity reshapes bone marrow progenitors, stromal niches, and downstream myelopoiesis.

Obesity also raises broader questions about the integration of metabolism with immunity. DCs act as sentinels, sampling their environment to shape T cell responses. If their metabolic fitness is compromised, this has knock-on effects for anti-tumour responses, antiviral defence and vaccine efficacy. These results place myeloid cells at the centre of the immunometabolic interface, illustrating a mechanism by which obesity shapes immune defence.

Furthermore, Yanan Hu and Svetoslav Chakarov review the diverse roles of eosinophils in metabolic health. Traditionally associated with type 2 immunity, eosinophils are now recognized as regulators of adipose tissue homeostasis [7]. In mice, they promote the conversion of white to beige adipocytes: a process that enhances thermogenesis and energy expenditure, thereby limiting weight gain and protecting against insulin resistance through IL-4 and IL-13 production. Loss of adipose eosinophils, in turn, worsens metabolic dysfunction. Yet, in obesity, the same eosinophil-derived cytokines can drive pathological fibrosis, particularly in the liver. In humans, the picture remains complex. While brown fat in infants is a key site of thermogenesis, the contribution of beige fat in adults remains uncertain, particularly in modern environments where external heating reduces the need for endogenous heat production. Clinical studies reflect this ambiguity: eosinophilia associates with metabolic syndrome in some contexts, but with protection from type 2 diabetes in others, depending on tissue and disease stage.

Taken together, these findings underscore two broader points: adipose tissue is not merely inert fat storage but a dynamic immune environment, and eosinophils embody a functional tension. While critical for parasite clearance, they also operate in metabolic tissues, sometimes providing protection and sometimes driving pathology, a theme that defines much of myeloid biology.

In a comprehensive review article, Alexander Mildner, Ki-Wook Kim, and Simon Yona revisit and challenge the long-held view of monocytes as simple macrophage precursors. Monocytes are thought to come in two flavours, with current nomenclature classing these as ‘classical’ and ‘non-classical’. While classical monocytes can fulfil their function as precursors by differentiating into homeostatic tissue macrophages, they are plastic and in certain contexts are inflammatory effector cells in their own right, or give rise to macrophages with exceptional wound-healing capacity. Non-classical monocytes, by contrast, are more terminally differentiated and act as steady-state sentinels, patrolling the vasculature and maintaining vascular integrity. The authors also discuss recent findings demonstrating that classical monocytes can arise through different progenitors and how this may impact their fate and function in tissue [8]. In metabolic diseases such as atherosclerosis and obesity, classical monocytes infiltrate adipose tissue and arteries, giving rise to specialized macrophages that balance lipid handling and inflammation. In autoimmunity, they mediate both tissue damage and repair, while in cancer, monocyte-derived macrophages adopt multiple phenotypes that influence angiogenesis, immune suppression, and antigen presentation.

These studies highlight monocytes as adaptable first responders whose functions are tightly shaped by environmental cues. Their plasticity makes them attractive therapeutic targets, but it also presents challenges, as interventions that suppress pathological activity may inadvertently impair essential repair functions. Understanding the developmental routes and transcriptional networks that underlie monocyte diversity will be key to future therapies.

Neutrophils similarly have undergone a conceptual transformation, once seen as short-lived effector cells with limited capacity, they are now recognized as transcriptionally dynamic. Huw Thomas, Steven Edwards and Helen Wright demonstrate that human neutrophils mount distinct gene programmes in response to different stimuli [9]. GM-CSF induces broad cytokine networks, TNFα drives NF-κB signalling in neutrophils and promotes their survival, while interferons activate antiviral pathways. This is coupled with treatment-specific changes in surface receptor expression. Notably, strong expression of negative regulators suggests that neutrophils carry intrinsic braking mechanisms to prevent runaway inflammation.

The recognition that neutrophils are context-responsive has major implications. In chronic inflammatory disease, prolonged survival and transcriptional reprogramming may perpetuate pathology. In cancer, tumour-associated neutrophils adopt diverse states that promote progression and immune evasion [10]. Neutrophils are therefore not simple foot soldiers of innate immunity but versatile regulators whose functional output is tailored to environment and stimulus.

As highlighted above, technological advances have led to reassessment of myeloid cell diversity. This also applies to mast cells and basophils, which were long viewed as simple effector granulocytes of type 2 immunity. Through release of granule components via high-affinity IgE receptors (FcεRI) and the production of IL-4, IL-5, IL-13, these play a central role in orchestrating type 2 responses, but they are more multifaceted than previously thought (Fig. 1).

**Figure 1.**
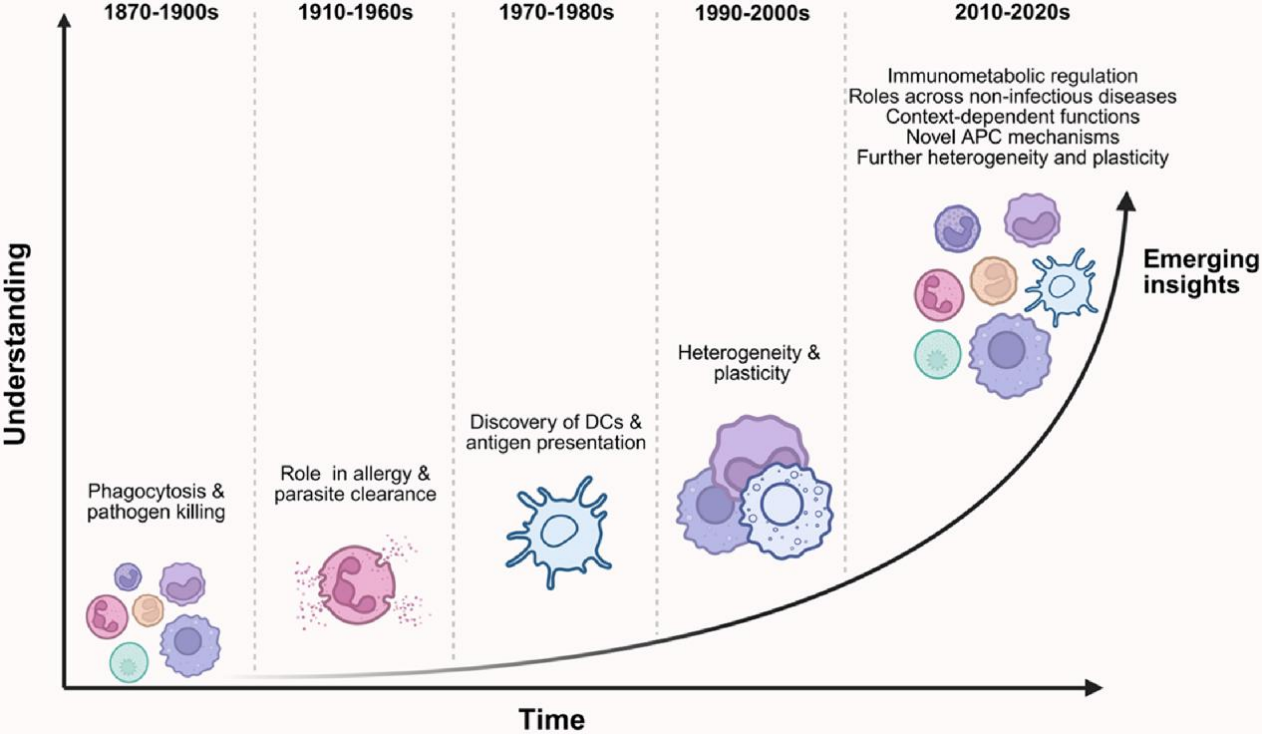
Evolution of our understanding of myeloid cells. Illustrative timeline summarizing the historical and conceptual advances in myeloid cell biology. In the late 19th to early 20th centuries, myeloid cells were first recognized for their phagocytic activity and role in pathogen killing. By the mid-20th century (1910–1960s), their contributions to allergy and parasite clearance were defined. The 1970–1980s marked a turning point with the discovery of DCs and antigen presentation, facilitated by advances in flow cytometry that allowed detailed analysis of rare populations. In the 1990–2000s, new animal models and the application of bulk transcriptomic approaches highlighted the remarkable heterogeneity and plasticity within the myeloid compartment. More recently (2010–2020s), single-cell RNA-sequencing, spatial transcriptomics, and other high-resolution technologies have revealed further layers of diversity, context-dependent functions, novel antigen presentation mechanisms, and immunometabolic regulation. Together, these advances illustrate the reappraisal of myeloid cells from simple effector cells to dynamic regulators of tissue and immune homeostasis across health and disease. Created in BioRender. Bankole, S. (2025). https://BioRender.com/7pao9um.

Mast cells are now emerging as important modulators of adaptive responses. Chi-Ching Tung, Abhay Rathore and Ashley St. John review evidence that mast cells can act as antigen-presenting cells (APCs) [11]. Unlike professional APCs, they acquire antigen via Fc receptor-mediated uptake or alternative pathways, with cytokines shaping uptake and presentation. They can express MHCII and co-stimulatory molecules, activate T cells, and influence memory and regulatory T cell responses. *In vivo*, mast cells can skew immunity toward Th1, Th2, or Treg outcomes, interact with γδ T cells (a subset of T cells with a distinct T cell receptor that responds rapidly to stress or infection) and NKT cells (lymphocytes that bridge innate and adaptive immunity) and promote cancer progression. These observations highlight mast cells as versatile immune regulators, whose outcomes depend on tissue context, microenvironmental signals, and immune history.

First described in 1879 but long neglected, basophils are now being redefined. Jiyeon Park and Suk-Jo Kang describe how technological advances reveal their evolutionary conservation and functional diversity. Beyond degranulation, basophils produce cytokines, influence antigen presentation, and shape tissue responses [12]. Ontogeny studies suggest multiple progenitor pathways and developmental flexibility. The existence of lung tissue resident basophils has also been shown through single-cell RNA-sequencing. Compared with circulating basophils, these exhibit a distinct transcriptional profile that is suggested to drive tissue-specific functions. The persistence of basophils across vertebrates underscores their essential roles as versatile regulators rather than redundant effectors.

The reappraisal of basophils and mast cells also prompts a reconsideration of allergic disease. Along with eosinophils, they evolved to help combat helminth infections. However, in environments where such infections are rare, their effector functions often manifest as allergic pathology. This illustrates a broader theme, evolutionary strategies that once conferred survival advantage can become maladaptive in modern contexts.

Together, these studies showcase the breadth and remarkable adaptability of myeloid cells. Once cast as merely innate effectors, myeloid cells are now recognized as highly heterogeneous, context-dependent regulators of immunity, metabolism, and tissue integrity during health, disease and tissue regeneration. Monocytes, neutrophils and basophils display remarkable flexibility, eosinophils balance adipose health and fibrosis, DCs integrate metabolic cues to tune responses, and mast cells are capable of bridging innate and adaptive systems in a manner distinct from professional APCs. Importantly, immune-mediated diseases illustrate how ancient strategies can become maladaptive in modern environments.

In an era defined by declining infectious burden but rising chronic disease, myeloid biology has re-emerged at the forefront of immunology. By embracing their heterogeneity, we can now appreciate myeloid cells not as one-dimensional effectors but as dynamic regulators whose evolutionary conservation reflects indispensable roles across health and disease. The challenge ahead is to translate this recognition into clinical insight. Just as checkpoint blockade reshaped cancer immunology by targeting T cell pathways, harnessing myeloid cell heterogeneity could open therapeutic opportunities in cancer, infection, inflammation, and metabolic disease [13]. The studies in this issue collectively argue that the future of immunology will not be written by adaptive immunity alone. Rather, it will depend on understanding how myeloid cells, our oldest immune allies, continue to shape human health in the twenty-first century.

## Data Availability

There are no new data associated with this article. No new data were generated or analysed in support of this research.

## Abbreviations

APC: Antigen-presenting cell
DC: Dendritic Cell
FcεRI: High-affinity IgE receptor
GM-CSF: Granulocyte-macrophage colony-stimulating factor
MHCII: Major histocompatibility complex class II
NF-κB: Nuclear factor kappa-light-chain-enhancer of activated B cells
NKT cell: Natural killer T cells
Th1/Th2: T helper type1/type 2 cells
TNFα: Tumour necrosis factor alpha
Treg: Regulatory T cell
γδ T cell: Gamma delta T cells
